# Case Report: A novel IRF2BP2 mutation in an IEI patient with recurrent infections and autoimmune disorders

**DOI:** 10.3389/fimmu.2023.967345

**Published:** 2023-06-07

**Authors:** Yiwen Pan, Guoguo Shang, Jing Li, Yuwen Zhang, Jianying Liu, Yuan Ji, Jing Ding, Xin Wang

**Affiliations:** ^1^ Department of Neurology, Zhongshan Hospital, Fudan University, Shanghai, China; ^2^ Department of Pathology, Zhongshan Hospital, Fudan University, Shanghai, China; ^3^ Center for Excellence in Brain Science and Intelligence Technology, Chinese Academy of Sciences, Shanghai, China; ^4^ The State Key Laboratory of Medical Neurobiology and Ministry of Education Frontiers Center for Brain Science, The Institutes of Brain Science and the Collaborative Innovation Center for Brain Science, Fudan University, Shanghai, China

**Keywords:** inborn errors of immunity, whole genomic sequencing, metagenomic sequencing, immune monitoring, IRF2BP2

## Abstract

**Introduction:**

Inborn errors of immunity (IEI) are a heterogeneous group of disorders characterized by increased risk of infections, autoimmunity, autoinflammatory diseases, malignancy and allergy. Next-generation sequencing has revolutionized the identification of genetic background of these patients and assists in diagnosis and treatment. In this study, we identified a probable unique monogenic cause of IEI, and evaluated the immunological methods and pathogenic detections.

**Methods:**

A family with a member with a clinical diagnosis of IEI was screened by whole genomic sequencing (WGS). Demographic data, clinical manifestations, medical history, physical examination, laboratory findings and imaging features of the patient were extracted from medical records. Comprehensive immune monitoring methods include a complete blood count with differential, serum levels of cytokines and autoantibodies, T-cell and B-cell subsets analysis and measurement of serum immunoglobulins. In addition, metagenomic sequencing (mNGS) of blood, cerebrospinal fluid and biopsy from small intestine were used to detect potential pathogens.

**Results:**

The patient manifested with recurrent infections and autoimmune disorders, who was eventually diagnosed with IEI. Repetitive mNGS tests of blood, cerebrospinal fluid and biopsy from small intestine didn’t detect pathogenic microorganism. Immunological tests showed a slightly decreased level of IgG than normal, elevated levels of tumor necrosis factor and interleukin-6. Lymphocyte flow cytometry showed elevated total B cells and natural killer cells, decreased total T cells and B-cell plasmablasts. WGS of the patient identified a novel heterozygous mutation in *IRF2BP2* (c.439_450dup p. Thr147_Pro150dup), which was also confirmed in his father. The mutation was classified as variant of uncertain significance (VUS) according to the American College of Medical Genetics and Genomics guidelines.

**Conclusion:**

We identified a novel *IRF2BP2* mutation in a family with a member diagnosed with IEI. Immune monitoring and WGS as auxiliary tests are helpful in identifying genetic defects and assisting diagnosis in patients with clinically highly suspected immune abnormalities and deficiencies in inflammation regulation. In addition, mNGS techniques allow a more comprehensive assessment of the pathogenic characteristics of these patients. This report further validates the association of IRF2BP2 deficiency and IEI, and expands IEI phenotypes.

## 1 Introduction

Inborn errors of immunity (IEI), previously referred to as primary immunodeficiency and immune dysregulation disorders (PID or PIDD), are a heterogeneous group of disorders that result from defects in immune system development and function (1, 2). Despite individual rarity, the estimated overall prevalence of IEI is approximately 1:1200-2000 (3, 4). The current classification consists of 485 genetic disorders, categorized into ten tables with subtables segregating groups of disorders into overlapping phenotypes in the 2022 update of the International Union of Immunological Societies (IUIS) (5, 6).

The affected patients present variable clinical manifestations, and increased susceptibility to a broad range of pathogens is the most common. Recently, an increasing diversity of autoimmune, autoinflammatory, malignant and allergic phenotypes have also been recognized (7). Other signs like a family history, failure to thrive, lymphopenia, hypogammaglobulinemia and poor response to prolonged use of antibiotics are also among the alarm bells of suspected IEI (8). IEIs may present at any age and the age of onset of symptoms is highly variable. Most patients had symptoms before the age of 16 (9). However, some patients are diagnosed in adulthood, either due to a lack of awareness preventing childhood diagnosis or because of delayed onset in adulthood (10).

As the discovery of new IEIs has been occurring at an impressive rate, 55 novel monogenic defects and 1 phenocopy have been added in the 2022 update of the IUIS (5). In particular, common variable immunodeficiency (CVID) is the most common symptomatic IEI but only 10–30% can be explained by a monogenic cause (11). IFN regulatory factor-2 binding protein 2 (IRF2BP2) is one of 20 known genes associated with CVID phenotypes, however knowledge about the role of IRF2BP2 for immune dysregulatory so far is limited (12).

In this study, we identified a novel IRF2BP2 mutation, which may be the probable unique monogenic cause of IEI. Immunological methods and pathogenic detections as auxiliary tests were evaluated in the patient.

## 2 Methods

In view of the diversity of clinical manifestations, timely and accurate diagnosis requires a high index of suspicion and specialized laboratory investigations. Diagnostic methods involve a simple blood count, flow cytometry, measurement of serum immunoglobulin levels, assessment of serum specific antibody titers in response to vaccine antigens, neutrophil function assays, stimulation assays for cytokine responses, and complement studies. Since infectious diseases are the predominant manifestation, new technologies such as metagenomic sequencing (mNGS) have been applied to detect pathogens (13). Simultaneously, the majority of these patients are deemed to have gene defects affecting the immune system. However, in most cases, the genetic background of the disease remains unidentified. The advent of next-generation sequencing (NGS) technologies has revolutionized the identification of genetic underpinnings of these patients and assists in diagnosis (14, 15). NGS is capable of high-throughput genomic sequencing with high sensitivity, from exonic (protein-coding) analysis to the entire genome in the form of targeted gene panels (TGPs), whole exome sequencing (WES), and whole genome sequencing (WGS). It can effectively discover new molecular defects, pathways of the diseases, pattern of inheritance, expand gene-disease phenotypes, and identify new gene-disease associations (16).

### 2.1 Participants and data collection

A family with a member with a clinical diagnosis of IEI and his parents was selected for molecular genetic testing. Demographic data, clinical manifestations, medical history, physical examination, laboratory findings and imaging features of the patient were extracted from medical records. The study was approved by the ethics committee of Zhongshan Hospital and has been performed in accordance with the ethical standards as laid down in the 1964 Declaration of Helsinki and its later amendments. A written informed consent was obtained from the patient’s parents since the patient had cognitive impairment and psychiatric symptoms.

### 2.2 Laboratory investigations

Immune monitoring methods include a complete blood count with differential, serum levels of cytokines and autoantibodies, T-cell and B-cell subsets analysis (with flow cytometry analysis), and measurement of serum immunoglobulins (using turbidimetry/nephelometry and enzyme-linked immunosorbent assay) (17, 18). In addition, due to recurrent fever of the patient, mNGS of blood, cerebrospinal fluid and biopsy from small intestine were used to detect unbiased thousands of pathogens. Therefore, we have a more comprehensive assessment of immunodeficient patient by considering both pathogen and immunity.

### 2.3 Next generation sequencing and mutation analysis

Genomic DNA was extracted from whole peripheral blood samples obtained from the participants for whole genomic sequencing (WGS). DNA samples were randomly disrupted by the ultrasonic high-performance sample processing system. The WGS and confirmatory Sanger sequencing methods were carried out according to a pipeline published previously (19). The classification of variant pathogenicity was based on the American College of Medical Genetics and Genomics (ACMG) guidelines (20, 21).

## 3 Case report and results

### 3.1 Timeline

**Figure 1:**
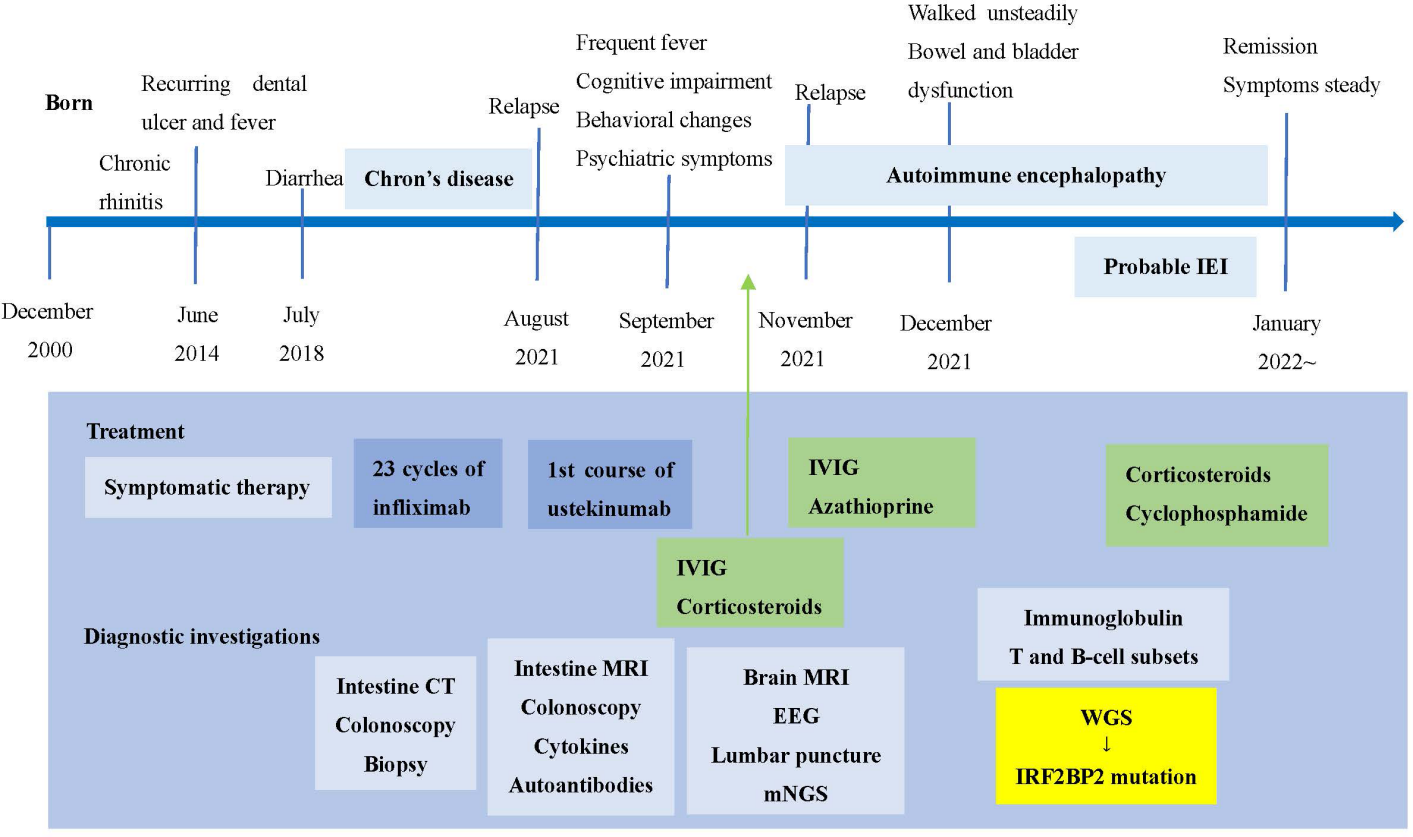
Timeline of clinical events, diagnostic investigations and treatment. IEI, inborn errors of immunity; IVIG, intravenous immunoglobulin; mNGS, metagenomic sequencing; WGS, whole genome sequencing.

The timeline of clinical events, diagnostic investigations and treatment is presented in **Figure 1**.

### 3.2 Clinical presentations of the patient and his family members

**Figure 2:**
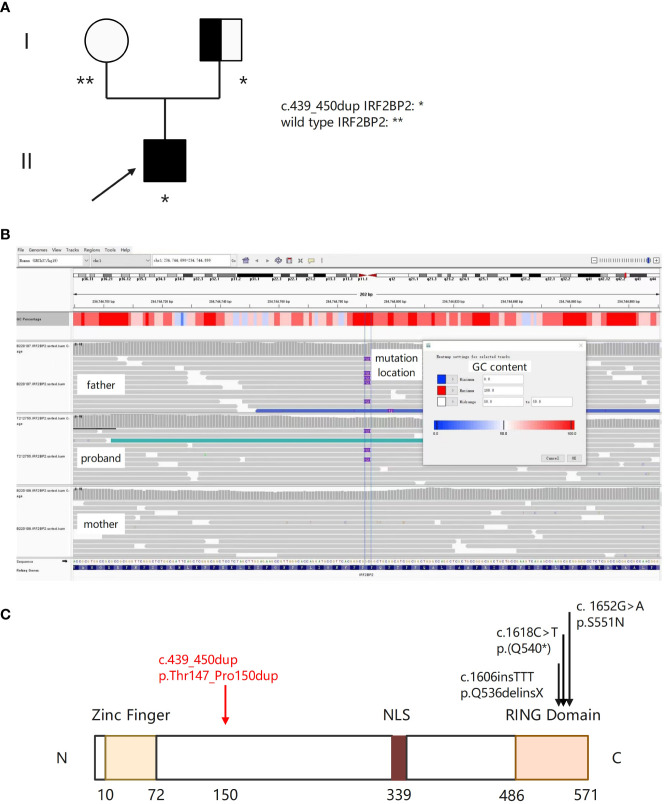
A novel IRF2BP2 mutation in a family with a member diagnosed with IEI. **(A)** Family tree with a novel heterozygous c.439_450dup mutation in the patient and his father. The proband is indicated by the arrow. Unfilled circle represents unaffected female, and the half-filled square represents unaffected male harboring the IRF2BP2 c.439_450dup variant. **(B)** Integrative Genomics Viewer (IGV) showing insertion of ACGCCGCAGCCG at position c.450 in exon 1 of IRF2BP2 in the proband and his father, and wild type sequence in his mother. **(C)** Schematic of the IRF2BP2 protein with an N-terminal Zinc Finger domain, a NLS (nuclear localization signal), and a C-terminal RING domain. The numbers indicate the amino acid positions. The c.439_450dup mutation results in duplication of amino acids (threonine, proline, glutamine, and proline) between position 147 and 150 (denoted by the red arrow). Black arrows indicate mutations of the other three published IRF2BP2 patients.

The patient is a 22-year-old man with chronic rhinitis history, who experienced recurring dental ulcer and fever since the age of 14, which could be ameliorated after antibiotics therapy. The symptoms worsened at age 18, and he also presented chronic diarrhea. Colonoscopy and biopsy findings in our hospital supported the diagnosis of Crohn’s disease-like lesion. Intravenous infusion of infliximab was conducted 23 times in the past three years and had a good effect. His father had chronic diarrhea in his childhood but recovered well after growing up, and he complained of no symptoms now. His mother, grandparents and other families are unaffected (**Figure 2A**).

### 3.3 Clinical progression of the patient

Unfortunately, the patient’s clinical symptoms, intestine imaging and colonoscopy deteriorated, and he received the first course of ustekinumab treatment on September 4, 2021. Three days later, he manifested frequent fever, cognitive impairment, behavioral changes, and psychiatric symptoms. Repetitive mNGS tests of blood samples and cerebrospinal fluid showed no evidence of bacterial, viral or mycotic infection. Initial brain magnetic resonance imaging (MRI) disclosed inflammatory changes in bilateral hippocampal areas. In addition, no underlying malignancy was detected. Lumbar puncture showed an increased intracranial pressure, and CSF analysis presented with pleocytosis and elevated protein content (**Table 1**). Cell based assay of autoantibodies associated with autoimmune encephalitis were negative in serum and CSF. We considered autoantibody-negative but probable autoimmune encephalitis (AE) (22), and initiated immunosuppressive therapy. The patient responded well.

**Table 1:** Laboratory investigations of the patient.

Date of test	On admission	After IVIG therapy	On discharge
WBC (3.50-9.50*10^9^/L)	11.25	9.70	10.07
Absolute neutrophil counts (1.8-6.3*10^9^/L)	8.6	6.9	8.0
Neutrophils (40.0-75.0%)	76.3	71.3	79.1
Absolute lymphocyte counts (1.1-3.2*10^9^/L)	1.4	1.9	1.2
Lymphocytes (20.0-50.0%)	12.0	19.8	12.0
Hb (130-175g/L)	134	128	148
PLT (125-350*10^9^/L)	158	217	161
CSF findings			–
Leukocytes, /mm^3^	88	25	–
Erythrocytes, /mm^3^	4	4	–
Protein (0.15-0.45g/L)	0.83	0.84	–
Glucose (2.5-4.5mmol/L)	2.3	3.0	–
IgG (8.60-17.40g/L)		9.18	7.45
IgG4 (0.03-2.00g/L)		0.12	–
IgA (1.00-4.20g/L)		1.96	2.25
IgM (0.30-2.20g/L)		0.82	0.74
IgE (<200IU/mL)		169	226

However, his symptoms recurred with fever and abdominal distension. We carried out immunological tests thoroughly and started the second course of intravenous methylprednisolone and IVIG, accompanied by oral azathioprine (AZA). Nevertheless, situation deteriorated again. We organized a multidisciplinary team discussion, and physicians achieved consensus on diseases of IEI. We started a more intensive immunosuppressive therapy with cyclophosphamide. On the day of discharge, a low dose of oral steroids (prednisolone) was maintained, the health condition of the patient improved steadily. The patient did adhere to the suggested treatment and followed up regularly.

### 3.4 Immunological investigations

On admission, a complete blood count showed elevated total leukocyte counts and absolute neutrophil counts (**Table 1**). Absolute lymphocytes were normal but the percentage was decreased. He was screened for autoantibodies, of which antinuclear antibody, anti-β2-glycoprotein 1 antibody, anti-SS-A antibody, and anti-mitochondrial M2 isoform antibody were positive.

Cytokines measurement showed elevated serum levels of tumor necrosis factor (TNF) and interleukin-1β (IL-1β) in almost all tests. Of note, we did not have record of our patient’s immunoglobulin levels prior to the first IVIG. Immunological evaluation showed normal levels of serum immunoglobulin (IgA, IgM, IgG and IgE) within one month after 2 cycles of IVIG, and a slightly decreased level of IgG than normal on discharge (**Table 1**). Lymphocyte flow cytometry showed elevated total circulating B cells (CD19+), normal nature killer (NK) cells and decreased T cells. More extensive immunophenotyping revealed a reduction in the proportions of B-cell plasmablasts (CD19+CD27+CD38+) and T follicular helper cells.

### 3.5 Identification of Novel *IRF2BP2* mutation by whole genome sequencing

In order to identify genetic etiology of the patient, we performed whole genome sequencing (WGS). Purified genomic DNA from this patient was subjected to WGS that utilized the Illumina paired‐end sequencing approach. Bioinformatics processing of the sequencing data and variant filtering were performed following the workflows. We attempted Sanger sequencing several times but did not obtain consistent results. Lastly, causative variants with functional impact relating to the clinical and immunological features of him were validated using Integrative Genomics Viewer (IGV). We obtained the same result by switching to a different NGS platform. Disease inheritance was confirmed by sequencing his parents’ DNA when available. WGS of the patient identified a novel heterozygous c.439_450dup p. Thr147_Pro150dup mutation in exon 1 of *IRF2BP2* (Mendelian Inheritance in Man (MIM) 615332; NM_182972.2), which was also confirmed in his father (**Figures 2A–C**). It results in duplication of amino acids (threonine, proline, glutamine, and proline) between position 147 and 150 (PM4). This domain was different from previous published IRF2BP2 mutations (**Figure 2C**). The mutations in the patient and his father were then screened in the latest catalog of genes known to be potentially mutated in IEI and public genomic databases. According to the databases of ESP, gnomAD and 1000 Genomes, the highest population frequency of this variant locus is zero (PM2). The variant is rare and is not found in population databases such as Clinvar and HGMD at the time of reporting. The mutation was classified as variant of uncertain significance (VUS) according to the ACMG guidelines based on the following criteria: PM4 (protein length change, **Figure 2C**), PM2 (variant absent from gnomAD database) and PP2 (a novel mutation which may be the probable cause of IEI). Eventually the patient was considered IEI based on the newest diagnostic criteria proposed by the European Society for Immunodeficiencies (ESID) (1).

## 4 Discussion

In this study, we identified a novel mutation in *IRF2BP2* in a patient with clinical diagnosis of IEI, which was also present in his father. The patient manifested with recurrent infections and autoimmune disorders since the age of 14, and symptoms got worse from adulthood.

Whilst increased risk of infections is a well-known IEI manifestation, currently other presenting clinical features of autoimmunity and immune dysregulation are taken seriously. There are various types of autoimmune manifestations, like hematological, endocrine, gastrointestinal, dermatological, and rheumatological disorders. Central nervous system (CNS) involvement is rare in IEI, especially in the form of autoimmune encephalitis (23). Therefore, it is essential yet particularly difficult to make proper diagnosis of autoimmune symptoms of IEI in CNS and differential diagnoses between other neurological disorders. The diagnosis of IEI is challenging and requires a high suspicion as well as reliable immunological evaluations and molecular evidence. Current molecular tests for CNS infectious disease are usually pathogen-specific that clinicians select relevant tests according to the symptoms of patients, which poses a challenge when novel or unexpected pathogens emerge. In contrast, mNGS can provide a comprehensive view of pathogens in a given sample, which enables the detection of novel and rare causative pathogens in the diagnosis of unexplained encephalitis. Since the disorders of IEI are genetically driven, most patients had symptoms and diagnosed before adulthood. However, in reality many IEIs present as “routine” infections (sinuses, ears or lungs) and are misdiagnosed or undetected. In addition, previous studies reported an even longer diagnosis delay in IEI patients with autoimmunity (24). Meanwhile, it is important to realize that secondary causes of immunodeficiency need to be ruled out. The patient had been diagnosed with Crohn’s disease and received the first course of ustekinumab treatment just before the onset of autoimmune neurological defects. The temporal association made it reasonable to consider an adverse effect of ustekinumab neurotoxicity. So far, clinical trials and real-world studies have reported very few ustekinumab-related neurologic adverse events, and all of them are characteristically reversible with discontinuation of the medication (25–27). In our patient, neurological symptoms didn’t get remission and even deteriorated after drug discontinuation. Consequently, ustekinumab should not be the chief culprit but probable specific trigger of the autoimmune manifestations of the patient.

Here we identified a novel heterozygous c.439_450dup p. Thr147_Pro150dup mutation in exon 1 of *IRF2BP2*. The *IRF2BP2* belongs to the IRF2BP family, and acts as an important new transcriptional cofactor in different biological systems (12). Recent studies reported that *IRF2BP2* has a role in macrophage regulation and lymphocyte activation, highlighting its function in immune responses (28, 29). It acts as a negative regulator of the NFAT (nuclear factor of activated T cells) transcription factor and plays a role in the differentiation and/or survival of memory B cells and plasmablasts (30). The protein-coding gene locates on chromosome 1q42.3 in humans and has 2 exons, producing three alternatively spliced proteins. *IRF2BP2* protein is composed of an N-terminal zinc finger and a C-terminal RING domain, however the function of which are not fully understood (31). Although IRF2BP2 deficiency is mentioned in classifications of monogenic CVID, only three families with autosomal dominant inheritance pattern have been described to date with missense and nonsense variants in IRF2BP2 (32–34). Keller etal (32). reported the first family with an autosomal dominant pattern of CVID, characterized with hypogammaglobulinemia and infections, but also psoriasis, colitis and type 1 diabetes. The phenotypic spectrum was finally identified with the *IRF2BP2* c.1652G>A p.S551N mutation, which impaired the plasmablast differentiation of B cells and immunoglobulin secretion *in vitro*. The phenotype in the second family was an IPEX (Immune dysregulation, polyendocrinopathy, enteropathy, X-linked) -like disease (35). This phenotypic spectrum was finally identified with the *IRF2BP2* c.1606insTT p.Q536delinsX mutation. Julia Ko¨rholz etal (32). reported another IRF2BP2-deficient patient with recurrent respiratory infections, colitis and rheumatoid arthritis. WES revealed a heterozygous *de novo* nonsense variant in IRF2BP2, c.1618C>T p.(Q540*). Several features of this patient’s phenotype match those of the patients described earlier. However, he developed severe CNS disorders as another sign of immune dysregulation.

In our case, the mutation results in duplication of amino acids between position 147 and 150, and this domain was different from previous published IRF2BP2 mutations. This was likely a rare variant within the genes databases, and was classified as VUS according to the ACMG guidelines. The mode of inheritance still remained obscure. We attempted Sanger sequencing several times but did not obtain consistent results. The possible reasons for this are as follows: 1) The insertion sequence contains repetitive sequences, and Sanger sequencing is not ideal for detecting insertions in repetitive regions because sequencing enzymes may slide in the repeat region, causing interruptions or frameshifts in the repeat sequence. 2) The insertion sequence has a high GC content within the ACGCCGCAGCCG, which may cause difficulties in denaturation during the sequencing reaction. We obtained the same result by switching to a different NGS platform. First, the mutation also occurred in his father, who didn’t have the clinical phenotype of IEI. Unfortunately, the father refused to perform complete laboratory tests, for he complained of no symptoms currently. Hypothetically it is due to gene penetrance, this is because these variants generally only make a small contribution to the multifactorial etiology of the condition (35). Another example is that TACI mutations are known to cause CVID, however 1% of the healthy population carry these mutations too. The common hypothesis is that TACI mutations are not fully penetrant and additional factors contribute to the development of CVID (36). Second, we didn’t perform further experimental studies that can certify the genetic variant impairs or alters expression or function of the *IRF2BP2* protein. Third, correlation between clinical features and the potential causative variants in this patient was assessed through a comprehensive literature search. However, the causal relationship between the mutation and clinical phenotype haven’t been confirmed by further functional validations. When possible, rescuing functional defect by reconstitution with the wildtype gene, or *via* a relevant animal phenotype will be needed to support the pathogenicity of the mutation (37).

Immunological evaluation showed normal levels of serum immunoglobulin within one month after IVIG therapy, and a slightly decreased level of IgG than normal after three months. Therefore, the diagnosis of CVID couldn’t be made. Therapeutic strategies include immunoglobulin supplement, immunosuppressive therapy for autoimmune manifestations, symptomatic treatment and close surveillance for the additional comorbidities. Long time follow-up is certainly required as the disease often relapsed and also for the revision of the classification of IEI. Despite the application of WGS technology for the diagnosis of IEI, there are still some problems to be solved. Since the databases are not perfect, the analysis may not be comprehensive. In addition, the report usually takes a long time, resulting in the lack of timely guidance for diagnosis and treatment. Future studies should also consider the contribution of environmental triggers and the potential for epigenetic effects.

## 5 Conclusion

We identified a novel *IRF2BP2* mutation in a family with a member with recurrent infections and autoimmune disorders, who was eventually diagnosed with IEI. This was probably the monogenic cause of the patient. This report further validates the association of IRF2BP2 deficiency and IEI, and expands IEI phenotypes. Immune monitoring and WGS as auxiliary tests are helpful in identifying genetic defects and assisting diagnosis in patients with clinically highly suspected immune abnormalities and deficiencies in inflammation regulation. In addition, mNGS techniques allow a more comprehensive assessment of the pathogenic characteristics of these patients. The management of infection and autoimmunity in patients with IEI requires special considerations because dysregulation of the immune system along with persistent inflammation impair the process of diagnosis and treatment. In the future, these techniques can further be translated into therapeutic targets for precision medicine.

## Data availability statement

The data presented in the study are deposited in National Genomics Data Center (https://ngdc.cncb.ac.cn/search), accession number HRA004688.

## Ethics statement

The studies involving human participants were reviewed and approved by the ethics committee of Zhongshan Hospital. The patients/participants provided their written informed consent to participate in this study. Written informed consent was obtained from the participant/patient(s) for the publication of this case report.

## Author contributions

XW, JD, YJ, GS and YP initiated and designed the study. YP and JLi collected and analyzed the clinical data. YZ and JLiu supervised and coordinated the data collection and the conduct of the study. JD, YJ, GS and YP drafted and revised the manuscript. All authors contributed to the article and approved the submitted version.
